# Neuroblastoma Mass Screening—What Can We Learn From It?

**DOI:** 10.2188/jea.JE20160038

**Published:** 2016-04-05

**Authors:** Kota Katanoda

**Affiliations:** Center for Cancer Registries, Center for Cancer Control and Information Services, National Cancer Center, Japan

**Keywords:** neuroblastoma, mass screening, early detection of cancer

In this issue of the *Journal of Epidemiology*, Ioka et al reported a study on the national mass screening program for neuroblastoma in Japan.^1^ The mass screening program for neuroblastoma was started in Kyoto City in 1973 and expanded as a national mass screening program in the mid-1980s. The screening was carried out by collecting urine samples from 6-month-old infants for the measurement of catecholamine metabolites (vanilmandelic acid and homovanillic acid). This method was widely accepted by parents having a new baby: the participation rate in 2001 was approximately 90%,^2^ which is much higher than the current participation rates in cancer screening programs for adults.

This screening method for neuroblastoma was developed in Japan.^3^ A study reported in *Cancer* in 1984 showed that the survival rate of neuroblastoma patients increased from 17.1% to 72.2% after the introduction of the mass screening program in Kyoto City.^3^ This must have encouraged many pediatricians, who had encountered many miserable outcomes in children. An epidemiologist might wish that this study could have been done with population-based mortality as the outcome. However, given that the annual number of deaths from this disease was <100 in the entire nation, the sample size might have been insufficient even if the city residents had been followed for decades.^4^

In the 1990s, descriptive epidemiological studies in Sapporo and Osaka examined the effects of mass screening for neuroblastoma by comparing the mortality rates between the post- and pre-screening periods. The study in Sapporo showed a reduction in mortality after the introduction of mass screening,^5^ whereas the study in Osaka reported that the screening program minimally affected mortality.^6^ There had also been a vigorous debate in clinical settings regarding the spontaneous regression of neuroblastoma detected by mass screening.^7^^–^^11^

In 2002, two historical reports were published in the same issue of the *New England Journal of Medicine*.^12^^,^^13^ The two studies, which were conducted in Germany and Canada, compared the mortality from neuroblastoma between screened and unscreened populations. Both studies showed that the neuroblastoma screening programs did not reduce mortality. In response to these findings, the Japanese Ministry of Health, Labour and Welfare (MHLW) organized a special committee in May 2003 to reconsider the rationale for the mass screening program for neuroblastoma. Three months later, the committee concluded that the program should be tentatively halted.

During the same period, a study group funded by the MHLW compared the participants and nonparticipants in the mass screening to evaluate the effect of mass screening on neuroblastoma mortality in Japan.^4^ The results revealed that mortality was reduced by 72% in the participants compared to nonparticipants. However, nonparticipants in the mass screening accounted for only 10% of the total study population. The possibility remained that the nonparticipants were likely to have a higher mortality not only from neuroblastoma but also from other causes. The study group planned to address this issue by comparing the all-cause mortality between participants and nonparticipants. Unfortunately, this additional study was not realized because the MHLW did not allow access to death certificates for this purpose.^4^ Although the reason for the discrepancy between results of overseas studies and those of the Japanese study remained unknown, the committee supported the cessation of the program. During the same period, and even after the decision of the committee, inconsistent epidemiological results continued to be reported.^14^^–^^16^

In the current issue, Ioka et al^1^ used data from the population-based Osaka Cancer Registry to compare neuroblastoma incidence and mortality across three periods: pre-screening, partial or systematic screening, and after cessation of screening. Their study covered a longer observation period than a previous paper in this journal.^17^ The main finding by Ioka et al was that there was no increase in mortality after the cessation of mass screening. Notably, they also examined changes in the incidence, stage distribution, and prognostic factors of neuroblastoma and found no substantial difference after the cessation of the program, except for a decrease in the overall incidence rate and the proportion of neuroblastomas identified at early stages.

What can we learn from the history of mass screening for neuroblastoma in Japan? The final report of the committee noted the need to thoroughly evaluate the effectiveness of a new screening method before implementing screening as a public health policy.^18^ However, we find numerous cases not necessarily following this principle, such as prostate cancer screening using prostate-specific antigen, human papilloma virus screening, and thyroid ultrasound examination in Fukushima. Several leading journals have recently addressed this issue in features titled “Too Much Medicine”^19^ and “Less is More”.^20^ Overuse of medical intervention may be a globally common problem. Ideally, evidence from randomized controlled trials should accumulate before introducing a screening program for a particular disease.^21^^,^^22^ In practice, however, interventions are not always initiated based on sufficient scientific evaluation. The issue of neuroblastoma mass screening will not be the last case requiring reconsideration of the rationale of a medical intervention that is already widely used in practice. In such cases, a well-designed observational study alone can provide the scientific basis for judgment. The study by Ioka et al in this issue is a good example of how epidemiology can play a role in real-world decision-making.
